# Maize Response to Low Temperatures at the Gene Expression Level: A Critical Survey of Transcriptomic Studies

**DOI:** 10.3389/fpls.2020.576941

**Published:** 2020-09-29

**Authors:** Paweł Sowiński, Jan Fronk, Maciej Jończyk, Marcin Grzybowski, Piotr Kowalec, Alicja Sobkowiak

**Affiliations:** ^1^Department of Plant Molecular Ecophysiology, Faculty of Biology, Institute of Plant Experimental Biology and Biotechnology, University of Warsaw, Warszawa, Poland; ^2^Department of Molecular Biology, Faculty of Biology, Institute of Biochemistry, University of Warsaw, Warszawa, Poland

**Keywords:** acclimatization, gene interaction network, moderately low temperatures, severe cold, *Zea mays* L., chilling

## Abstract

Maize is a cold-sensitive plant whose physiological reactions to sub-optimal temperatures are well understood, but their molecular foundations are only beginning to be deciphered. In an attempt to identify key genes involved in these reactions, we surveyed several independent transcriptomic studies addressing the response of juvenile maize to moderate or severe cold. Among the tens of thousands of genes found to change expression upon cold treatment less than 500 were reported in more than one study, indicating an astonishing variability of the expression changes, likely depending on the experimental design and plant material used. Nearly all these “common” genes were specific to either moderate or to severe cold and formed distinct interaction networks, indicating fundamentally different responses. Moreover, down-regulation of gene expression dominated strongly in moderate cold and up-regulation prevailed in severe cold. Very few of these genes have ever been mentioned in the literature as cold-stress–related, indicating that most response pathways remain poorly known at the molecular level. We posit that the genes identified by the present analysis are attractive candidates for further functional studies and their arrangement in complex interaction networks indicates that a re-interpretation of the present state of knowledge on the maize cold-response is justified.

## Introduction

Maize exhibits numerous features interesting from both the basic and applied points of view (Walbot, 2009). It is highly diversified at the genomic level, which likely was the key to its spectacular dispersal within a few millenia only into environments highly distinct from that of its domestication (Tenaillon and Charcosset, 2011). Despite that successful adaptation to different climate conditions, some limitations to maize cultivation remain, the susceptibility to low temperatures being the major one in the temperate climate. Here, at germination and during the following shift from heterotrophic to autotrophic, growth maize is often subjected to prolonged cold periods, while at juvenile growth (up to the V6 growth stage), short cold spells may occur. Long-lasting cold at a very early growth stage mostly affects the rate of development of new leaves (Ben-Haj-Salah and Tardieu, 1995; Giaufret et al., 1995; Tardieu, 2003) and formation of the photosynthetic apparatus (Sowiński et al., 2005; Grzybowski et al., 2019). In contrast, at the V3–V5 growth stages a few cold days with minimal temperatures around 5°C during the day can be sufficient to cause photoinhibition (Fryer et al., 1995), chlorosis, membrane damage, and eventually necrosis or even plant death (Janowiak and Markowski, 1994). These fundamentally different physiological responses warrant distinguishing two types of cold stress: moderately low temperatures (12–15°C) and severe cold (below ca. 8°C) (Stamp, 1984; Marocco et al., 2005; Frascaroli and Revilla, 2018). This distinction, however, is not absolute, since when severe cold is preceded by a several-day period of low temperatures above 12°C, the disturbances are less profound, which is a sign of cold-acclimation (Leipner et al., 2000; Sobkowiak et al., 2016). The growth and development are still slowed down, but no permanent injury ensues. Some view an enhancement of the maize acclimatability as a route to a further improvement of its performance in the temperate climate (Sobkowiak et al., 2016; Szalai et al., 2018).

Notably, while the physiological response of maize to low temperatures is well understood, its molecular foundations are not. Until recently, only a handful of genes and molecular mechanisms likely engaged in that response were identified using classical approaches (reviewed in: Marocco et al., 2005; Leipner and Stamp, 2009; Frascaroli and Revilla, 2018). A true quantitative breakthrough came with the advent of transcriptomics allowing numerous papers on that subject to be published. These studies have now reached a sufficiently advanced stage to warrant a critical summing up with an attempt at drawing wider conclusions.

## A Search for Key Cold-Affected Maize Genes

In an attempt to identify key molecular events in the maize response to suboptimal temperatures, we searched the databases shown as Supplementary materials of relevant transcriptomic studies for genes reported by more than a single study. Only projects concerning the juvenile phase of growth (V2–V5) and, with few exceptions, limited to the leaf/shoot were considered. The plant materials investigated were highly diversified, from well-established classical representatives of the main heterotic groups to novel lines from across the modern maize cultivation range (see **Table S1** in **Supplementary Materials** for details). The different experimental approaches used in those studies offered hope that any genes found as shared between such diverse projects would be related to the pivotal features of the maize response to low temperatures rather than reflecting the particulars of a given experiment. The studies were divided into two groups according to the cold severity (moderate or severe). In each group, one project reporting the highest number of differentially expressed genes (DEGs) was taken as a reference with which other datasets were compared. To allow the comparison between papers, the gene terminology was uniformized according to the maize genome version AGPv3.

### Moderately Low Temperatures

Four transcriptomic studies of the maize response to moderate cold have been published (Trzcinska-Danielewicz et al., 2009; Sobkowiak et al., 2016; Avila et al., 2018; Szalai et al., 2018; **Table S1**). Overall, seven genotypes were investigated; two projects reported over 10,000 DEGs each, and two only found several hundred DEGs. The study with the widest scope (three lines) and the highest number of DEGs was used as a reference (Sobkowiak et al., 2016). For a broader perspective, data from *Miscanthus* x *giganteus*, a cold-tolerant C4 grass, were also included (Spence et al., 2014).

Only 226 DEGs were shared between the reference and at least one other study (cmDEGs, common for moderate cold, **Table S2**). Twice as many cmDEGs (117) showed consistent down-regulation than up-regulation (51), while 58 exhibited an inconsistent behavior between projects. These latter genes seem worthy of inspection as their contrasting response could reflect a complex (e.g., diurnal) pattern of expression changes “frozen” upon sampling, the timing of which differed between the projects studied. Alternatively, the differences in expression could simply reflect the different plant ages/organs used in individual projects. In view of the strong dominance of gene down-regulation found at all consistency levels (i.e., the cmDEGs common to two, three or all four projects), one could argue that the maize reaction to a moderate cold simply consists in a slowing down of all processes, including gene expression. However, the very low percentage of the DEGs common to two or more projects among all the DEGs reported in those projects argues for a particular importance of the cmDEGs.

**Figure 1:**
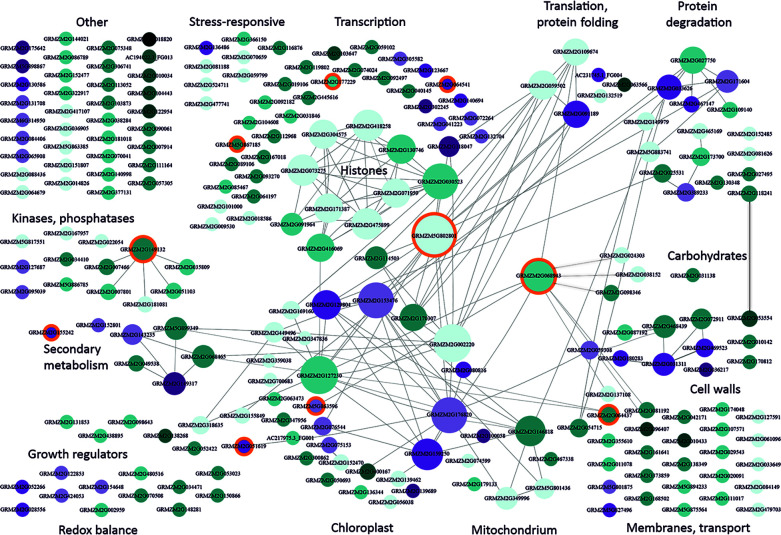
Functional classification and interactions of maize genes responding to moderately low temperatures. cmDEGs (**Table S2**; see text for definition) were assigned function based on gene description and available characteristics (Gramene, MaizeGDB) and grouped manually into categories according to main function or organellar localization. Interactions between genes were found using Cytoscape v. 3.7.1. (Shannon et al., 2003). Genes are represented by circles with GRMZM numbers. The size of the circle is proportional to the number of gene partners (interactors) and interactions are indicated by lines aimed at the circle centre. Cyan marks down-regulated genes and magenta – up-regulated ones. The intensity of colouring is proportional to the number of datasets reporting the gene as changing expression in moderate cold. Light blue depicts genes showing inconsistent behavior in different studies (i.e., down-regulated in one or more study and up-regulated in another study(ies)). Orange background marks genes changing also in response to severe cold and listed in **Supplementary Materials, Table S6**. This figure is also available in an interactive format as **Supplementary Figure S1**.

To identify biological relations among the cmDEGs, we created their interaction network using Cytoscape (Shannon et al., 2003). **Figure 1** shows a strikingly non-random functional distribution of these genes. The largest group among the cmDEGs comprised 43 genes related to transcription. It contained several genes encoding histones and other proteins related to chromatin condensation, numerous transcription factors, and several DNA or RNA binding proteins, including two RNA splicing factors. The genes encoding histones and other proteins related to chromatin status are engaged in a rich interaction network (each gene has several partners within this sub-group), but the most connected (26 partners) is GRMZM5G802801 [heat shock protein7; also defined as probable mediator of RNA polymerase II transcription subunit 37c (LOC103635762)]. It interacts with three other proteins related to transcription and also with members of the second-largest group of genes (27) related to chloroplast functioning.

As already mentioned, at the physiological level moderate chilling evokes a rather mild stress in maize, causing slowing down or cessation of growth. This is reflected in the present analysis as a general down-regulation of cmDEGs (apparent in **Figure 1** as a strong dominance of cyan over magenta). The widespread and mostly uniform changes in the expression of genes related to chromatin and transcription suggest that a global modification of the chromatin structure could underlie the general repression of transcription. Notably, repressed are genes related to auxin signalling (GRMZM2G155849, Protein AUXIN SIGNALING F-BOX 3; GRMZM2G098643, Putative auxin efflux carrier; GRMZM2G138268, Auxin-responsive protein IAA14; GRMZM2G438895, auxin-like 1 protein), while an enzyme responsible for gibberellin inactivation (GRMZM2G051619, gibberellin 2-oxidase6) is induced, all these changes being consistent with the physiological response, i.e., cessation of growth.

The chloroplast-related genes show a slightly less uniform response than the transcription-related ones, and the changes are less reproducible among the different studies. Several genes are strongly connected, with up to 22 partners, both within the group and with outside cmDEGs. The notion that cold affects the photosynthetic apparatus functioning in maize is widely accepted (Foyer et al., 2002). The decrease of photosynthetic activity observed in maize at moderately low temperatures is to some extent reflected in the transcriptomic data as numerous chloroplast-related genes are down-regulated. However, only one gene directly related to photosynthesis is affected, pyruvate orthophosphate dikinase4 (AC217975.3_FG001), a proposed key site for C4 regulation; its role in maize photosynthesis under cold conditions is not clear (Leipner and Stamp, 2009). On the other hand, several genes related to chloroplast functioning are up-regulated, mostly encoding proteins involved in the control of translation, which could herald an induction of cold-acclimation.

Several other genes deserve a mention owing to their consistency – or, conversely, inconsistency, with the physiological data. Thus, an involvement of the cell wall in the cold-acclimation of maize is well established (Sobkowiak et al., 2016; Bilska-Kos et al., 2017); consistently, two cell-wall-related cmDEGs were identified in nearly all studies: up-regulated GRMZM2G036217 (male sterile protein homolog1; ascribed to wax esters biosynthesis I pathway (CornCyc)) and down-regulated GRMZM2G053554 (Alpha-galactosidase 3). However, overall there are surprisingly few signs of acclimation, since other mechanisms postulated to be involved in cold acclimation, such as induction of ROS scavengers or remodelling of the photosynthetic apparatus, do not seem to be up-regulated. This discrepancy could be due to the fact that the studies analysed here used fairly short chilling periods, while substantial cold-acclimation is only achieved following prolonged cultivation of maize at a low temperature and, apart from an accumulation of pigments (Haldimann, 1998) , mainly involves slow processes such as ultrastructural modifications of the photosynthate transport path (Sowiński et al., 2003) and formation of a xeromorphic body shape (Verheul et al., 1996; Grzybowski et al., 2019).

### Severe Cold

Five studies reporting over 1,000 DEGs each, carried out on an aggregate of nine inbred lines, formed the core set of projects analysed here (Fernandes et al., 2008; Sobkowiak et al., 2014; Jończyk et al., 2017; Lu et al., 2017; Waters et al., 2017; **Table S1**). Among them the reference study (Sobkowiak et al., 2014) investigated two lines and found almost 8,000 genes changing expression in at least one line. Results of four additional RNA-seq studies reporting less than 1,000 DEGs each complemented the comparison (Shan et al., 2013; Aguilar-Rangel et al., 2017; Mao et al., 2017; Li et al., 2019).

**Figure 2:**
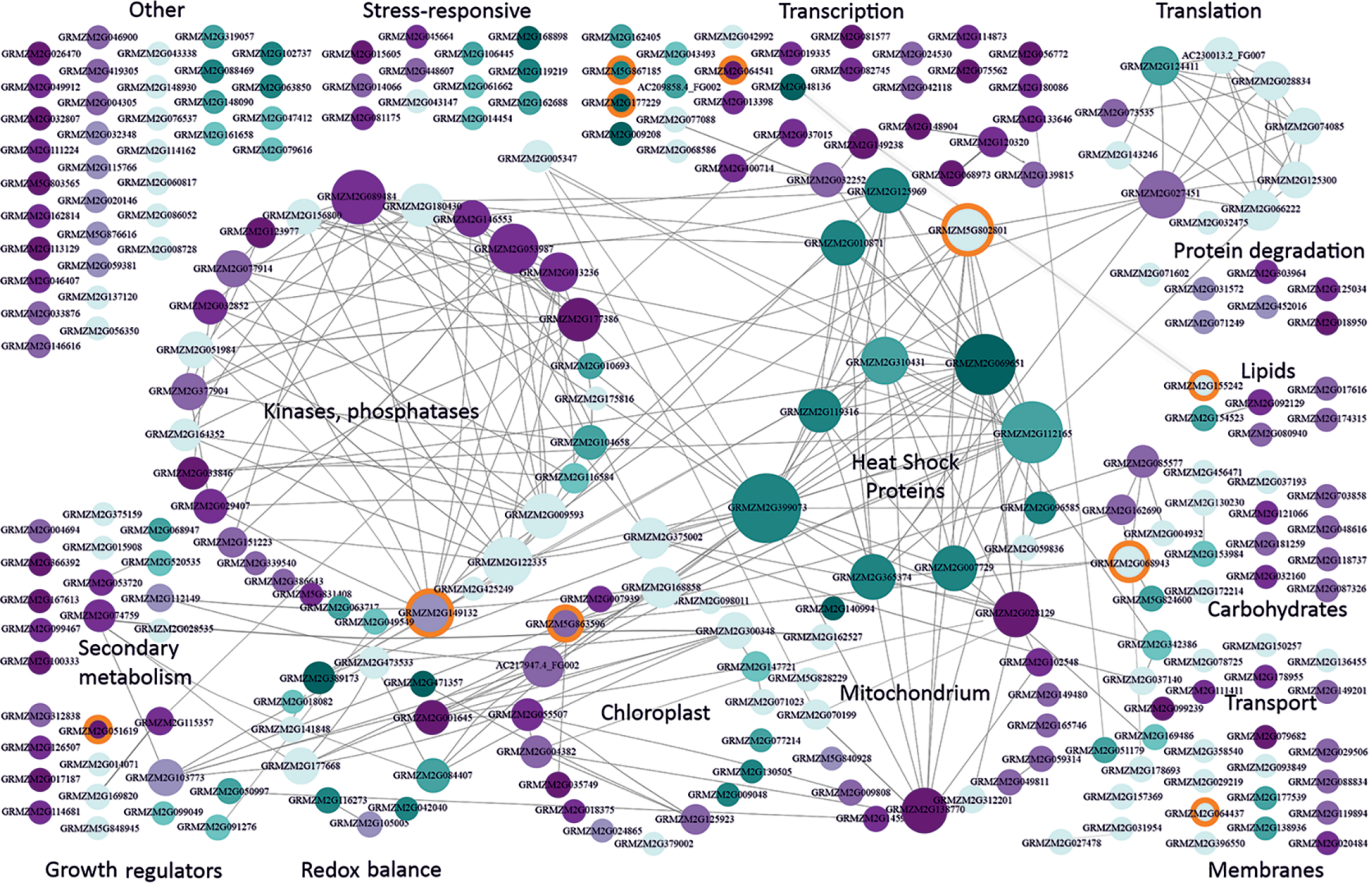
Functional classification and interactions of maize genes responding to severe cold. csDEGs (**Table S3**; see text for definition) were assigned function basing on gene description and available characteristics (Gramene, MaizeGDB) and grouped manually into categories according to main function or organellar localization. Interactions between genes were found using Cytoscape v. 3.7.1. (Shannon et al., 2003). Genes are represented by circles with GRMZM numbers. The size of the circle is proportional to the number of gene partners (interactors), and interactions are indicated by lines aimed at the circle center. Cyan marks down-regulated genes, and magenta marks up-regulated ones. The intensity of colouring is proportional to the number of datasets reporting the gene as changing expression in severe cold. Light blue depicts genes showing inconsistent behavior in different studies [i.e., down-regulated in one or more study and up-regulated in another study(ies)]. Orange background marks genes changing also in response to moderate cold and listed in **Supplementary Materials, Table S6**. This figure is also available in an interactive format as **Supplementary Figure S2**.

Two-hundred and fifty-seven DEGs were shared between the reference and at least one other study (csDEGs, common for severe cold, **Table S3**). In stark contrast to moderate cold, severe cold mainly caused an up-regulation of expression (124 up-regulated vs. 61 down-regulated csDEGs). The largest group (**Figure 2**) comprised genes related to transcription (33 csDEGs); they showed little or no connectivity except for three genes discussed later. The second-largest and highly connected group comprised genes encoding protein kinases and phosphatases, including several up-regulated members of the MAP-kinase pathway and also up-regulated type 2C protein phosphatases. MAP kinases and type 2C phosphatases counteract each other and take part in signal transduction in plants, including the ABA and stress signalling pathways (Fuchs et al., 2013). Also, calcium-dependent kinases, well known for their participation in stress signalling (Schulz et al., 2013) including cold, were found.

Considering the well-established fact that severe cold compromises photosynthesis in maize, likely through photoinhibition (Dolstra et al., 1994; Govindachary et al., 2004), it was unexpected that, in fact, not so many chloroplast-related csDEGs were found and that more than half were up-regulated. Only three genes were clearly related to the light phase of photosynthesis: GRMZM2G009048 (photosystem I N subunit 2) was down-regulated, and two peptidases, GRMZM2G024865 (Thylakoidal processing peptidase 1 chloroplastic) and GRMZM2G379002 (carboxyl-terminal-processing peptidase 2 chloroplastic), were up-regulated.

The number of mitochondria-related csDEGs was the same as that for chloroplasts, and they also were mostly up-regulated. Unlike chloroplasts, mitochondria have been mentioned in the context of cold stress in maize rather sparingly (Stewart et al., 1990; De Santis et al., 1999), therefore the present results seem noteworthy as they suggest a comparable participation of these two organelles in the maize cold-response. We would like to draw attention to two homologs of the mitochondrial AAA-ATPase ASD from *Arabidopsis thaliana* (GRMZM2G028129 and GRMZM2G138770) showing highly consistent behavior in all the studies analysed and a rich network of interactions. In *A. thaliana*, the ASD ATPase has been found to respond to low temperature in an ABA-dependent manner (Baek et al., 2011).

In contrast to the above groups showing predominant up-regulation, a small group of heat-shock proteins were exclusively down-regulated. These genes are also the most highly connected ones, both within the group and with other groups. Among their partners one finds HSF-transcription factor 4 (GRMZM2G125969) and HSF-transcription factor 24 (GRMZM2G010871), as well as heat shock protein7/probable mediator of RNA polymerase II transcription subunit 37c (GRMZM5G802801); these three genes were also down-regulated. Notably, the response of the heat-shock genes and their transcription-related partners was highly reproducible among most or even all the studies. This feature suggests a fundamental character of this response in severe-cold conditions. An up-regulation of HSPs is often observed as a key component of the plant response to abiotic stresses (Wang et al., 2004), one may thus hypothesize that the failure to ensure the production of properly folded proteins—as indicated by HSPs down-regulation and induction of protein degradation (**Figure 2**)—is a main cause of the maize sensitivity to severe cold.

## Discussion

The patterns of the transcriptomic changes in moderately low temperatures and in severe cold are fundamentally different and overall are in line with the respective physiological reactions of maize – simple slowing down of growth and development vs. profound and often irreversible damage. Moderate cold causes a general down-regulation of genes related to the transcription apparatus itself. In contrast, plants confronted with a life-threatening stress apparently activate diverse protective mechanisms, which manifests in the maize response to severe cold as induction of expression of genes related to major signalling pathways and also to the transcription machinery. However, the parallel down-regulation of a large group of genes related to protein folding, in particular heat shock proteins (HSPs), likely makes this response futile. Alternatively, the induction of different signalling pathways massively up-regulating transcription could reflect an unspecific “panic” alarm response to severe stress, ineffective in protecting the plant against the stress. This possibility is supported by the apparently counterproductive changes of the two major growth-regulating hormonal pathways as evident in **Figure 2**: up-regulation of auxin signalling in severe cold (in contrast to moderate cold, where it is down-regulated) and inactivation of gibberellins due to up-regulation of gibberellin 2-oxidase6.

### Candidate Critical Cold-Response Genes

In view of the different designs of individual projects analysed in the present study and their use of different maize lines we decided to use the least-stringent criterion possible and consider as noteworthy all genes that showed expression changes in at least two projects (cDEGs). However, it seems obvious that genes found to change expression in all (rather than just two) of the studies would likely represent a fundamentally important feature – consistently engaged in the cold (moderate or severe) response regardless of the maize genotype or experimental design. Relatively few such genes were found for either set of studies; they can be identified in **Figures 1**, **2** as the most darkly coloured ones and are listed in **Tables S4** and **S5**, respectively.

Strikingly, the vast majority of such potentially critical genes have never been mentioned in the literature in the context of cold stress in any plant species.

Among the 17 highly consistent DEGs found for the moderate-cold experiments (**Table S4**) only one has been reported earlier: Male sterile protein homolog 1 (GRMZM2G036217) was down-regulated in maize inbred line A661 which became albinotic upon cold treatment (Rodríguez et al., 2013). For severe cold, only four out of 30 highly consistent DEGs (**Table S5**) have ever been mentioned. Peroxidase 52 (GRMZM2G471357, down-regulated in maize) was found to be involved in preventing lipid peroxidation and maintaining leaf cell water potential, key cellular adaptations to severe-cold-tolerance in banana (He et al., 2018). NaCl stress protein1 (GRMZM2G015605, up-regulated in maize) has an *A. thaliana* ortholog AT3G05880.1 described in the TAIR database as induced by low temperature, dehydration, salt stress, and ABA (https://www.arabidopsis.org/servlets/TairObject?type=gene&name=AT3G05880.1). Remorin (GRMZM2G099239, up-regulated) has a maize paralog GRMZM2G081949 which was down-regulated after prolonged cold-treatment both at moderate and severe cold (Bilska-Kos et al., 2016), i.e., it responded in the opposite direction to GRMZM2G099239. Notably, it showed up-regulation in a cold-tolerant inbred line after short cold-treatment (ibid.). Finally, gibberellin 2-beta-dioxygenase (GRMZM2G051619), up-regulated in maize, was also up-regulated in severe-cold-treated cassava (An et al., 2012) and rice (Pan et al., 2011).

As discussed earlier, the maize responses to moderately low temperatures and to severe cold are markedly different at the physiological level and this is also clearly visible at the level of gene expression changes. Nevertheless, certain changes do link the two temperature regimes as evidenced by the presence of a set of 10 genes shared between the cmDEGs and csDEGs (**Table S6**); marked by orange background in **Figures 1**, **2**, and **Figure S1**, **S2**).

Remarkably, nine of these genes can be classed in just two highly coherent groups: signalling and gene expression. The remaining odd gene is alpha-amylase 3, up-regulated in both stress conditions, which is unexpected in view of starch accumulation (not hydrolysis) in cold-treated maize (Sowiński et al., 1999).

Of the five signalling-related genes only gibberellin 2-oxidase6 behaves consistently across projects; its up-regulation is compatible with growth cessation in the cold. The other genes react differently in different studies indicating their likely subtle regulation depending on experimental design or cold severity. Two of these genes are functionally related—proton *myo*-inositol cotransporter imports inositol, a core component of a family of secondary messengers, while low phytic acid1 converts *myo*-inositol into its hexakis phosphate. The participation of protein phosphatases (here, Probable protein phosphatase 2C 22) and trehalose (here represented by trehalose-6-phosphate synthase 1) in plant signalling has been postulated (Avonce, 2004; Komatsu et al., 2013). In contrast to the former group, the four gene-expression-related genes—two related to transcription and two to splicing, show fully consistent behavior across studies, indicating another, apart from gibberellin degradation—element common to the maize response to moderate and severe cold.

### Concluding Remarks

The present survey of transcriptomic studies highlights several previously unappreciated facts regarding the maize response to low temperatures. There is little overlap between the results of individual studies, as a vast majority of all gene expression changes are specific to the particular study. Apparently, both the biological material used (genotype, organ) and the experimental design affect the obtained results. However, despite this variability remarkably consistent patterns of gene expression changes can be identified by focusing on the results shared by several independent projects. These patterns are fundamentally different depending on the stress severity; overall down-regulation of expression of numerous groups of genes is observed upon moderate chilling, while prominent induction of diverse regulatory circuits ensues in severe cold. Also the genes responding to moderate cold are generally different from those reacting to severe cold and both groups deserve detailed studies to shed light on the specificity of the two responses.

On the other hand, the very small set of genes affected by moderate and severe cold alike may indicate the crux of the maize reaction to sub-optimal temperatures, and their investigation should help identify the most fundamental molecular mechanisms of this reaction. We find it particularly noteworthy that of only 10 such genes two are related to splicing, suggesting a pivotal role of this stage of gene expression in the stress response.

## Data Availability Statement

All datasets presented in this study are included in the article/**Supplementary Material**.

## Author Contributions

PS conceptualized the paper. PS and JF wrote the article. MJ carried out bioinformatical analyses. PS, MG, PK, and AS analyzed the raw data. All authors contributed to the article and approved the submitted version.

## Funding

This work was supported by grant 2017/27/B/NZ9/00995 from the National Science Centre (NCN), Poland.

## Conflict of Interest

The authors declare that the research was conducted in the absence of any commercial or financial relationships that could be construed as a potential conflict of interest.
