# Supplementary material for: Maize Response to Low Temperatures at the Gene Expression Level: A Critical Survey of Transcriptomic Studies
Source: Front Plant Sci. 2020 Sep 29;11:576941. doi: 10.3389/fpls.2020.576941 (PMC7550719; doi:10.3389/fpls.2020.576941)
Supplement: Supplementary file 1 [file Presentation_1.pptx]

## Slide 1
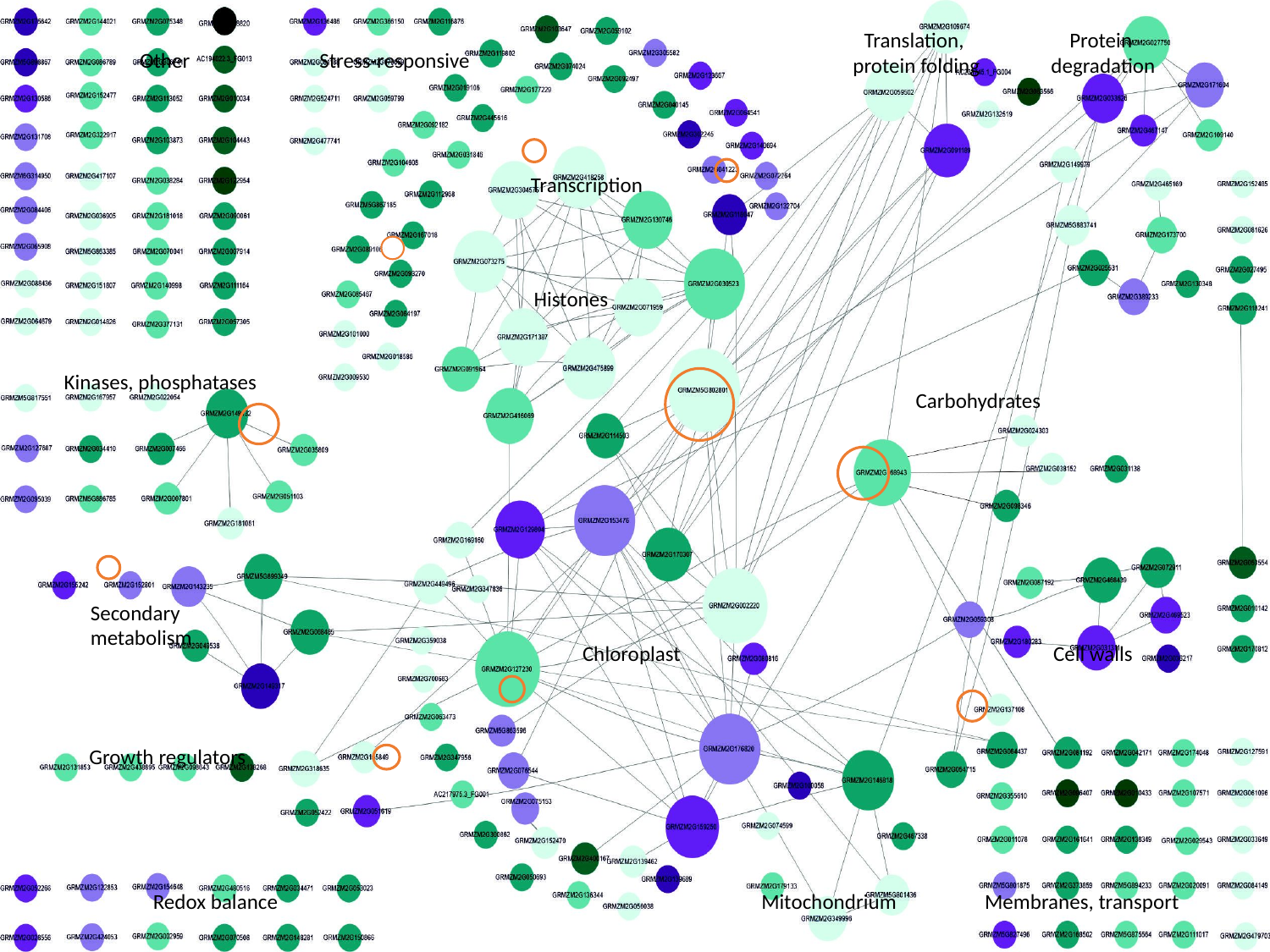

Protein
degradation
Translation,
protein folding
Other
Stress-responsive
Transcription
Histones
Kinases, phosphatases
Carbohydrates
Secondary
metabolism
Chloroplast
Cell walls
Growth regulators
Mitochondrium
Redox balance
Membranes, transport
