# Supplementary material for: Maize Response to Low Temperatures at the Gene Expression Level: A Critical Survey of Transcriptomic Studies
Source: Front Plant Sci. 2020 Sep 29;11:576941. doi: 10.3389/fpls.2020.576941 (PMC7550719; doi:10.3389/fpls.2020.576941)
Supplement: Supplementary file 2 [file Presentation_2.pptx]

## Slide 1
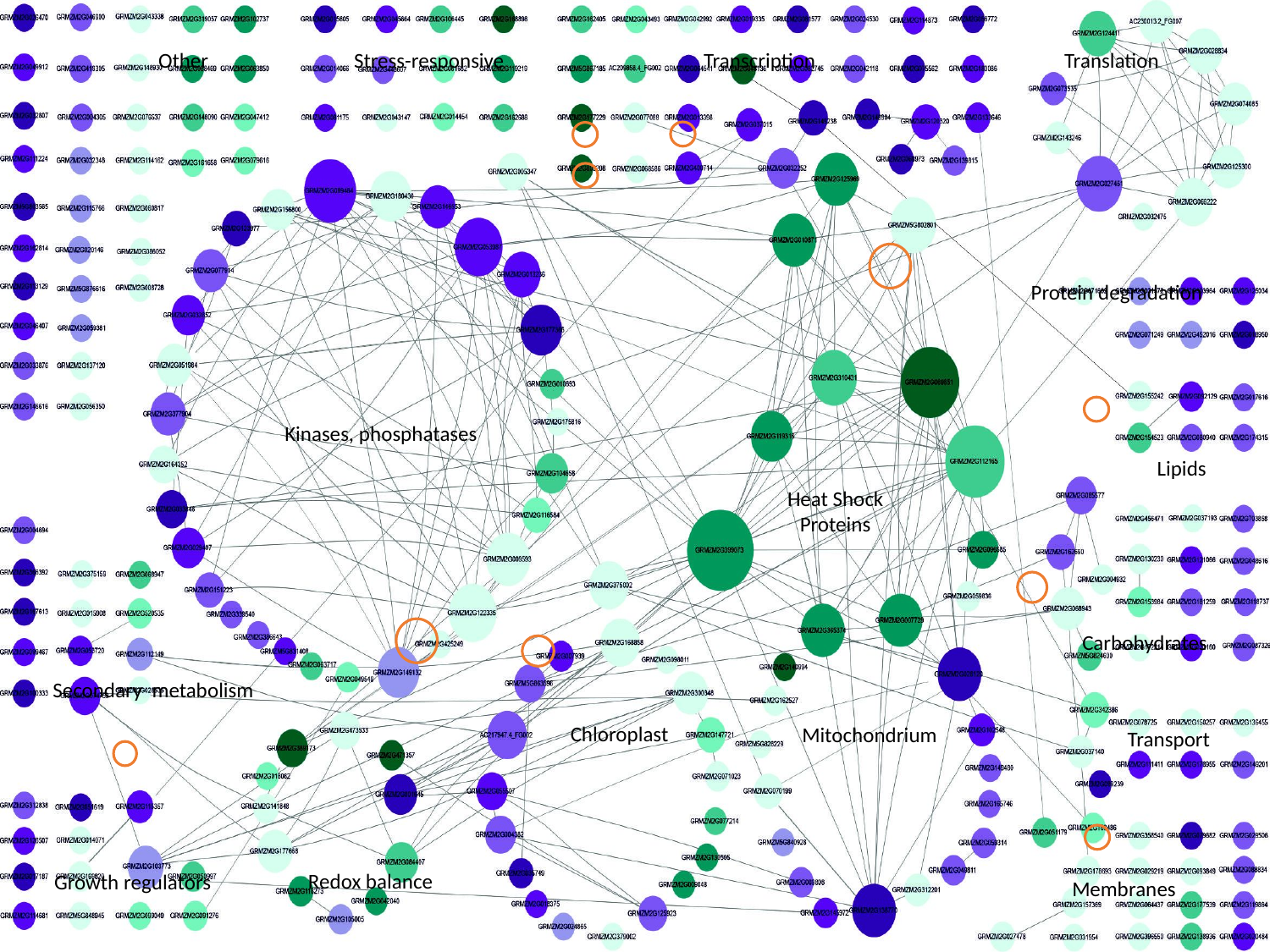

Other
Stress-responsive
Transcription
Translation
Protein degradation
Kinases, phosphatases
Lipids
Heat Shock
Proteins
Carbohydrates
Secondary metabolism
Chloroplast
Mitochondrium
Transport
Redox balance
Growth regulators
Membranes
