# Supplementary material for: Maize Response to Low Temperatures at the Gene Expression Level: A Critical Survey of Transcriptomic Studies
Source: Front Plant Sci. 2020 Sep 29;11:576941. doi: 10.3389/fpls.2020.576941 (PMC7550719; doi:10.3389/fpls.2020.576941)
Supplement: Supplementary file 3 [file Table_1.docx]

Table S1. Transcriptomic studies of the maize response to low temperatures

| Stress | Transcriptomic technique | Genotype/  material | Experimental | Reference |
| --- | --- | --- | --- | --- |
| Moderately  low temperatures | Microarrays, Maize Oligonucleotide Array Project, The University of Arizona, Tucson, 44K, one-chip version | Inbred lines S160, S50676, S68911, Poland | 14°/12°C,  38 h (10 h dark, 14 h light, 10 h dark, 4 h light), middle part of  third leaf blade at V3 | Sobkowiak *et al.*, 2016 |
|  | RNA-seq | Inbred lines  CG60, CG102, USA | 14°/2°C,  three days, second leaf  at V2 | Avila *et al.*, 2018 |
|  | Microarrays, Agilent Whole Corn Gene Expression Microarray 4 × 44K | Hybrid Nona, Hungary | 15°/13°C, three days,  leaf blade at V3 | Szalai *et al.*, 2018 |
|  | Microarrays, Maize Oligonucleotide Array Project, The University of Arizona, Tucson, 60K, two-chip version | Inbred line CM109, Germany | 14°/12°C,  28 h (14h – light, 10h – dark, 4h – light) | Trzcinska-Danielewicz *et al.*, 2009 |
| Auxiliary project | | | | |
|  | Microarrays, Agilent Whole Corn Gene Expression Microarray 4 × 44K | *Miscanthus × giganteus* | 14°/12°C, 14 days, midpoint of youngest fully expanded leaf | Spence *et al.*, 2014 |
|  | | | | |
| Severe cold | Microarrays, Maize Oligonucleotide Array Project, The University of Arizona, Tucson, 44K, one-chip version | Inbred lines ETH-DH7, ETH-DL3, Switzerland | 8°/6°C, 14 h (10 h dark, 4 h light), middle part of third leaf blade at V3 | Sobkowiak *et al.*, 2014 |
|  | Microarrays, Maize Oligonucleotide Array Project, The University of Arizona, Tucson, 44K, one-chip version | Inbred line CM109, Germany | 8°/6°C,  24 h (10 h dark, 14 h light), seven timepoints, middle part of third leaf blade at V3 | Jończyk *et al.*, 2017 |
|  | RNA-seq | Inbred lines B73, Mo17, PH207, Oh43, USA | 7°C, 16 h (4 h light, 8 h dark, 4 h light), above-ground organs,  14-day-old seedlings | Waters *et al.*, 2017 |
|  | RNA-seq | *Z. mays* ssp. *mexicana,* variety 8493,  China | 4°C, 12 h,  entire 13-day-old seedlings | Lu *et al.*, 2017 |
|  | Microarrays, Maize Oligonucleotide Array Project, The University of Arizona, Tucson, 60K, two-chip version | Inbred line B73, USA | 10°C, 24 h (final 8 h in the dark), entire seedlings | Fernandes *et al.*, 2008 |
| Auxiliary projects | | | | |
|  | RNA-seq | *Z. mays* spp. *saccharata*, inbred lines RC, C5,  China | 8°/5°C, five days, leaves and roots, V3 | Mao *et al.*, 2017 |
|  | RNA-seq | Elite inbred line Zheng58,  China | 4°C, 24 h, entire seedlings, V3 | Shan *et al.*, 2013 |
|  | RNA-seq | Maize highland landrace Palomero Toluqueño,  Mexico | 5°C, 1 h, leaves, 14-day-old seedlings | Aguilar-Rangel *et al.*, 2017 |
|  | RNA-seq | Inbred lines M54, 753F,  China | 4°C, 4 h and 24 h, leaves, V4 | Li *et al.*, 2019 |
