# Supplementary material for: Maize Response to Low Temperatures at the Gene Expression Level: A Critical Survey of Transcriptomic Studies
Source: Front Plant Sci. 2020 Sep 29;11:576941. doi: 10.3389/fpls.2020.576941 (PMC7550719; doi:10.3389/fpls.2020.576941)
Supplement: Supplementary file 6 [file Table_4.docx]

Table S4. Maize genes responding to moderately low temperatures reported in all four studies surveyed

| Gene symbol | Description |
| --- | --- |
| *Down-regulated* | |
| GRMZM2G122954 | NA |
| GRMZM2G104443 | NA |
| AC194022.3_FG013 | uncharacterized LOC100281482 |
| GRMZM2G010034 | uncharacterized LOC100277758 |
| GRMZM2G018820 | Glycerophosphodiester phosphodiesterase GDPD2* |
| GRMZM2G103647 | Basic leucine zipper 9 |
| GRMZM2G010433 | Transmembrane amino acid transporter family protein |
| GRMZM2G063566 | Ribonucleoprotein A1, A2/B1 homolog |
| GRMZM2G096407 | Amino acid permease 6 |
| GRMZM2G053554 | Alpha-galactosidase 3 |
| *Up-regulated* | |
| GRMZM5G898867 | NA |
| GRMZM2G175642 | NA |
| GRMZM2G118047 | HSF28 HSF type transcription factor |
| GRMZM2G100058 | Pentatricopeptide repeat-containing protein mitochondrial |
| GRMZM2G036217 | male sterile protein homolog1 |
| GRMZM2G139689 | L-aspartate oxidase chloroplastic |
| GRMZM2G302245 | regulator of chromosome condensation1 |

* - The gene also showed down regulation in cold-treated *Miscanthus × giganteus*

(Spence et al., 2014)
