# Supplementary material for: Maize Response to Low Temperatures at the Gene Expression Level: A Critical Survey of Transcriptomic Studies
Source: Front Plant Sci. 2020 Sep 29;11:576941. doi: 10.3389/fpls.2020.576941 (PMC7550719; doi:10.3389/fpls.2020.576941)
Supplement: Supplementary file 7 [file Table_5.docx]

Table S5. Maize genes responding to severe cold reported in at least five studies surveyed

| Gene symbol | Description |
| --- | --- |
| *Down-regulated* | |
| GRMZM2G048136 | Putative MYB DNA-binding domain superfamily protein |
| GRMZM2G009208 | rmr2 - required to maintain repression 2 |
| GRMZM2G140994 | Rhomboid-like protein 14 mitochondrial |
| GRMZM2G177229 | c3h40 - C3H-transcription factor 340 (Splicing factor U2af small subunit A) |
| GRMZM2G471357 | Peroxidase 52 |
| GRMZM2G168898 | hemoglobin2 |
| *Up-regulated* | |
| GRMZM5G803565 | NA |
| GRMZM2G032807 | NA |
| GRMZM2G113129 | NA |
| GRMZM2G017187 | Auxin response factor 6 |
| GRMZM2G035749 | Beta-amylase 3 chloroplastic |
| GRMZM2G026470 | Soluble inorganic pyrophosphatase; Uncharacterized protein |
| GRMZM2G015605 | nac1 - NaCl stress protein1 |
| GRMZM2G123977 | ankyrin repeat-containing protein putative expressed |
| GRMZM2G177386 | Protein phosphatase 2C 16 |
| GRMZM2G064541 | nactf66 - NAC-transcription factor 66 |
| GRMZM2G068973 | nactf23 - NAC-transcription factor 23 |
| GRMZM2G079682 | Beta-1,4-mannosyl-glycoprotein 4-beta-N-acetylglucosaminyltransferase |
| GRMZM2G051619 | Gibberellin 2-beta-dioxygenase |
| GRMZM2G056772 | gras14 - GRAS-transcription factor 14 |
| GRMZM2G075562 | Zinc finger protein CONSTANS-LIKE 9 |
| GRMZM2G081577 | B-box type zinc finger family protein |
| GRMZM2G100333 | Probable ethanolamine kinase |
| GRMZM2G028129 | AT5G40010, AAA-ATPase ASD mitochondrial |
| GRMZM2G138770 | AT5G40010, AAA-ATPase ASD mitochondrial |
| GRMZM2G001645 | 3-hydroxy-3-methylglutaryl-coenzyme A reductase putative |
| GRMZM2G149238 | TATA-binding protein1 |
| GRMZM2G366392 | S-adenosylmethionine decarboxylase proenzyme |
| GRMZM2G148904 | S-adenosyl-L-methionine-dependent methyltransferases superfamily protein |
| GRMZM2G099239 | Remorin |

Listed are genes reported in all five main studies or in four such studies and additionally in at least one auxiliary study.
