# Supplementary material for: Maize Response to Low Temperatures at the Gene Expression Level: A Critical Survey of Transcriptomic Studies
Source: Front Plant Sci. 2020 Sep 29;11:576941. doi: 10.3389/fpls.2020.576941 (PMC7550719; doi:10.3389/fpls.2020.576941)
Supplement: Supplementary file 8 [file Table_6.docx]

Table S6. Maize genes responding to both moderately low temperatures and severe cold

| Gene symbol | Description | Moderate cold* | | Severe cold* | |
| --- | --- | --- | --- | --- | --- |
| GRMZM5G867185 | SCL25B arginine/serine-rich splicing factor SCL25B transcript III | - | a, c, *e* | - | f, g, h, j |
| GRMZM2G177229 | c3h40 - C3H-transcription factor 340  (splicing factor U2AF putative expressed) | - | a, c | - | f, g, h, j, *n* |
| GRMZM5G802801 | heat shock protein7/Probable mediator of RNA polymerase II transcription subunit 37c | 0 | a, c (-)  b (+) | 0 | f, g, h, i (-)  j (+) |
| GRMZM2G068943 | trps1 - trehalose-6-phosphate synthase1 | - | a, b, c | 0 | f, g, j (-)  h (+/-)  i (+) |
| GRMZM2G064437 | proton myo-inositol cotransporter | - | a, b, c | 0 | f, g (-)  h, i (0)  j (+) |
| GRMZM2G149132 | Probable protein phosphatase 2C 22 | - | a, c | + | g, j |
| GRMZM2G155242 | lpa1 - low phytic acid1 | + | a, b, c | 0 | g, h, i (-)  f, j (+) |
| GRMZM2G051619 | ga2ox6 - gibberellin 2-oxidase6 | + | a, b, c | + | f, g, h, j, *k,* *l, n* |
| GRMZM5G863596 | alpha-amylase 3, chloroplastic | + | a, c, *e* | + | f, g, j |
| GRMZM2G064541 | nactf66 - NAC-transcription factor 66 | + | a, b, c | + | f, g, h, j, *k* |

This list represents the set common to cmDEGS and csDEGs (see text for definitions).

*”-“ and “+” indicate genes, respectively, down-regulated and up-regulated in all studies from a given treatment regime (moderate or severe cold), “0” indicates genes showing inconsistent behaviour in different studies from a given treatment regime.

References: a. Sobkowiak et al., 2016; b. Avilla et al., 2018; c. Szalai et al., 2018; d. Trzcińska-Danielewicz et al., 2009; *e. Spence et al., 2014*; f. Sobkowiak et al., 2014; g. Jończyk et al., 2017; h. Waters et al., 2017; i. Lu et al., 2017; j. Fernandes et al., 2008; *k. Mao et al., 2017; l. Shan et al., 2013; m. Aguilar-Rangel et al., 2017; n. Li et al., 2019*. Auxiliary datasets are indicated by italics.
